# Improved Voltage and Cycling for Li^+^ Intercalation in High‐Capacity Disordered Oxyfluoride Cathodes

**DOI:** 10.1002/advs.201500128

**Published:** 2015-06-12

**Authors:** Shuhua Ren, Ruiyong Chen, Emad Maawad, Oleksandr Dolotko, Alexander A. Guda, Viktor Shapovalov, Di Wang, Horst Hahn, Maximilian Fichtner

**Affiliations:** ^1^ Institute of NanotechnologyKarlsruhe Institute of TechnologyP.O. Box 364076021KarlsruheGermany; ^2^ Helmholtz Institute Ulm89081UlmGermany; ^3^ Institute of Materials ResearchHelmholtz‐Zentrum Geesthacht22607HamburgGermany; ^4^ Heinz Maier‐Leibnitz ZentrumTechnische Universität München85748GarchingGermany; ^5^ International Research Center–Smart MaterialsSouthern Federal University344090Rostov‐on‐DonRussia; ^6^ Joint Research Laboratory NanomaterialsTechnische Universität Darmstadt64287DarmstadtGermany

**Keywords:** disordered rock salt, intercalation cathodes, Li‐ion batteries, oxyfluoride

## Abstract

**New high‐capacity intercalation cathodes** of Li_2_V*_x_*Cr_1−*x*_O_2_F with a stable disordered rock salt host framework allow a high operating voltage up to 3.5 V, good rate performance (960 Wh kg^−1^ at ≈1 C), and cycling stability.

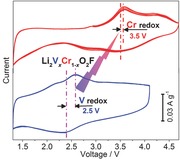

Lithium‐ion batteries as attractive power sources have contributed to the commercial success of portable electronics and are undergoing rapid expansion to applications in electric vehicles and smart‐grid technologies.^1^, ^2^ Although extensive studies have been devoted to improve the intercalation‐based cathode materials (polyanion compounds,^3^, ^4^, ^5^, ^6^ spinels,^7^, ^8^ and layered oxides,^9^, ^10^, ^11^ so far achieved energy storage capability is still not adequate to meet the demands of future markets. Structurally, electrode performance can often be deteriorated by unintended cation disordering/intermixing^12^, ^13^, ^14^, ^15^, ^16^, ^17^ and phase transition for these classic cathode materials.^18^, ^19^, ^20^, ^21^ Strategies to suppress the cation migration and phase change over cycling are considered in general to be crucial to improve the electrochemical performance.^22^, ^23^, ^24^ In contrast, upon cycling, structural transformation into disordered rock salt (DRS) has been found to favor a stable and reversible lithium intercalation storage, superior to that in the parent structure.^25^, ^26^, ^27^ To date, direct synthesis of several DRS oxides^28^, ^29^, ^30^, ^31^ has been reported. In the DRS structure, lithium and transition metal(s) distribute randomly at the same crystallographic site (4*a* Wyckoff site) with close‐packed oxygen (at 4*b* Wyckoff site) sublattice. Li‐excess in DRS oxides offers distinct macroscopic percolation pathways for lithium diffusion.^25^, ^32^ However, so far reported DRS materials are still not able to afford competitive capacity and energy density compared to the classic intercalation cathode materials. Development of new high‐performance electrode materials with large reversible capacity, high voltage, and rate capability remains a great challenge.

Recently, we have demonstrated that a DRS oxyfluoride Li_2_VO_2_F has a low lattice volume variation of only 3.3% for 1.8 Li^+^ intercalation.^33^ Lithium, occupying 2/3 of the cation sites in Li_2_MO_2_F, is highly mobile with diffusion channel spanning the entire structure.^32^ Unlike the principle of Li^+^ storage in a crystal framework with ordered Li^+^ site/layer, wherein Li^+^ diffusion can be easily impeded due to structural defects/changes (such as cationic relocation,^12^, ^13^, ^14^ antisite disorder,^15^, ^16^, ^17^ and layer slab narrowing^25^, ^32^), Li^+^ hopping in the lithium‐rich DRS structure is facile and relatively independent of the Li content upon charge/discharge.^25^, ^32^ Hence, such DRS framework is highly desirable for achieving high energy/power density. Nevertheless, the redox reactions based on vanadium occur at a relatively low voltage (2.5 V) in the DRS oxyfluoride system and the cyclability needs to be improved.^33^ Hence, it remains a great interest to survey alternative transition metals that can operate at higher voltages without compromising the reversible capacity and rate capability.

Herein, we report that the key performance parameters of average discharge voltage and cyclability can be improved through the substitution of chromium for vanadium in the dilithium DRS Li_2_V*_x_*Cr_1−*x*_O_2_F oxyfluoride cathodes. Participation of Cr (centered at 3.5 V) and V (at 2.5 V) redox couples and the reversible structural changes over cycling were evidenced by detailed characterizations. A maximum specific energy of ≈1140 Wh kg^−1^ was obtained for *x* = 0.2 at C/35 and room temperature. Attractively, high specific energy of ≈960 Wh kg^−1^ is still accessible even at 1 C rate.

A series of solid solution Li_2_V*_x_*Cr_1−*x*_O_2_F (*x* = 0, 0.2, 0.5, 0.8) materials were synthesized by a simple ball milling route. The disordered cubic rock salt structure (space group *Fm‐3m*) was identified for all synthesized materials (Figure S1, Supporting Information). **Figure** **1**a shows combined synchrotron X‐ray diffraction (SXRD) and neutron diffraction (ND) patterns for the representative sample of *x* = 0.2, which exhibits superior electrochemical performance with high specific capacities and high discharge voltages (discussed later). The obtained pattern data were converted to *d*‐spacing values for better comparison. Dashed lines indicate the diffraction peaks of cubic rock salt phase. Strong diffraction peaks of (200) and (220) from the rock salt structure are clearly evidenced in SXRD, whereas those of (111), (311), and (331) with high neutron reflection intensities were complementarily detected by ND. Rietveld refinements with the space group *Fm‐3m* gave a good fit of the experimental data (Figures S2 and S3, Supporting Information). Linear increase of lattice parameters with *x* values indicates a solid solution system following Vegard's law (Figure 1c). The increase of lattice constant with *x* is expected with the substitution of Cr^3+^ ions (0.615 Å) with larger V^3+^ ions (0.640 Å).^34^ Certain impurities from WC and Cr_2_O_3_ (about 12 wt% refined from SXRD pattern, Figure S3, Supporting Information) were observed. Cr K‐edge X‐ray absorption near‐edge structure (XANES) for the as‐prepared Li_2_V*_x_*Cr_1−*x*_O_2_F series showed similar spectrum compared to the reference Cr_2_O_3_, indicating that the Cr ions were trivalent and octahedrally coordinated (Figure S4, Supporting Information). High‐resolution transmission electron microscopy (HR‐TEM) indicated nanocrystalline character of as‐synthesized Li_2_V*_x_*Cr_1−*x*_O_2_F (Figure S5, Supporting Information). ^7^Li and ^19^F MAS NMR spectra showed broad resonance lines for both pristine *x* = 0.2 and *x* = 0.8 samples, indicating large distribution of chemical shifts in such DRS structure (Figure S6, Supporting Information).

**Figure 1:**
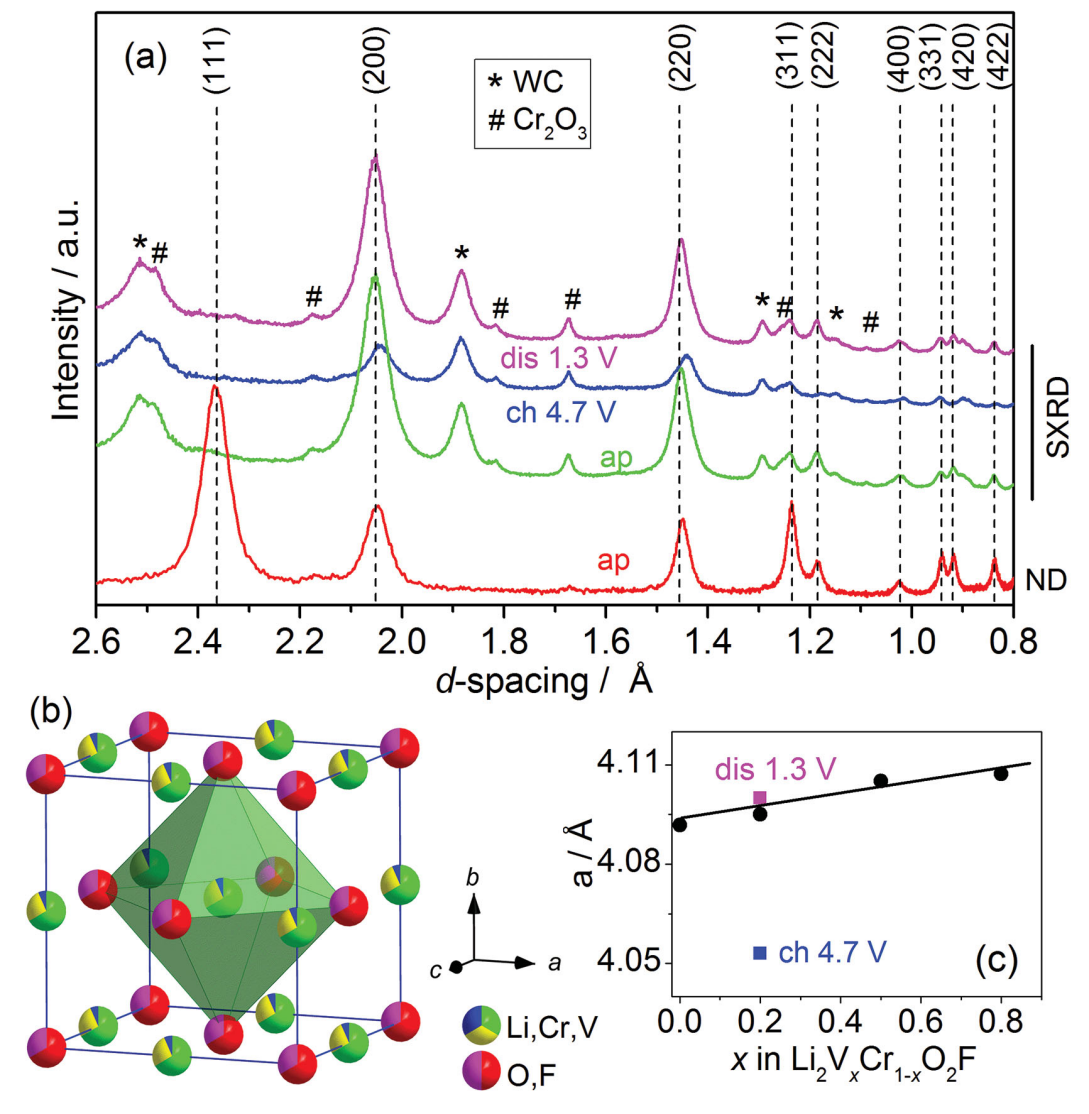
a) ND and SXRD patterns for as‐prepared Li_2_V_0.2_Cr_0.8_O_2_F and the corresponding charged (4.7 V) and discharged (1.3 V) samples; b) disordered rock salt unit cell for Li_2_V_0.2_Cr_0.8_O_2_F; c) variation of lattice parameters for rock salt Li_2_V*_x_*Cr_1−*x*_O_2_F (*x* = 0, 0.2, 0.5, 0.8), lattice parameters for charged (4.7 V) and discharged (1.3 V) samples of *x* = 0.2 are labeled as blue and pink color, respectively.

The involved redox couples of Cr and V can be well identified from cyclic voltammetry (CV) analysis (**Figure** **2**). CV curves at a scan rate of 0.05 mV s^−1^ for Li_2_V*_x_*Cr_1−*x*_O_2_F (*x* = 0, 0.2, 0.5, 0.8) are plotted in Figure 2a. With gradual increase of chromium, the main redox peaks shift from 2.5 to 3.5 V. The redox for V‐rich composition (*x* = 0.8) is centered at ≈2.5 V. The coexistence of both ≈2.5 and ≈3.5 V potentials becomes quite apparent for *x* = 0.5 and *x* = 0.2. For pure chromium oxyfluoride (*x* = 0), two successive oxidation voltages centered at about 3.6 and 4.3 V and a broad reduction peak centered at ≈3.5 V were observed. These may be attributed to the redox reaction of chromium. Furthermore, the Cr redox shows small polarization of only ≈0.05 V, compared to ≈0.2 V for V (Figure 2a). Thus, it can be deduced that both chromium and vanadium are electrochemically active and their redox couples participate in electrochemical delithiation/lithiation processes. Overall discharge voltages can be significantly enhanced by introduction of chromium. A kinetic study by CV analysis under varied scan rates (0.02–10 mV s^−1^) was performed. The cathodic peak current response is proportional to the square root of scan rate, indicating that Li^+^ storage in Li_2_V*_x_*Cr_1−*x*_O_2_F arises mainly from a solid‐state diffusion‐controlled intercalation mechanism.^33^, ^35^, ^36^ Further structural analysis to confirm the redox process will be discussed below.

**Figure 2:**
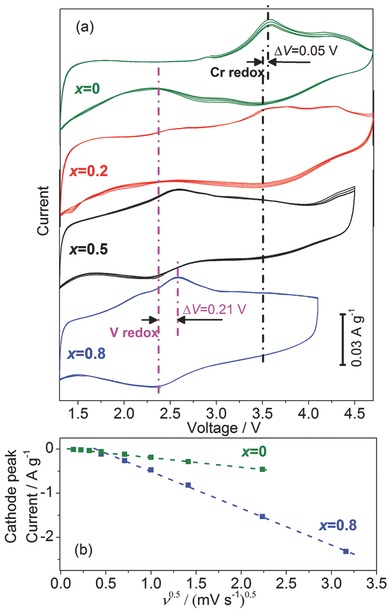
a) CV curves of Li_2_V*_x_*Cr_1−*x*_O_2_F (*x* = 0, 0.2, 0.5, 0.8) at 0.05 mV s^−1^ and 25 °C in varied voltage ranges; b) the plots of cathodic peak current versus the square root of sweep rate for *x* = 0 and *x* = 0.8.

Galvanostatic charge/discharge performance was studied for Li_2_V*_x_*Cr_1−*x*_O_2_F at room temperature and 13 mA g^−1^ (≈C/35). Varied voltage ranges were applied for different *x*. The performance dependence on cutoff voltages will be discussed below. Charge/discharge profiles in the first three cycles are shown in **Figure** **3**a–d. Differences between the first charge and discharge capacities for V‐rich oxyfluoride of *x* = 0.8 may arise from off‐stoichiometry.^33^ In comparison, for Cr‐rich oxyfluorides (*x* = 0, 0.2), certain side reactions at high voltages^37^, ^38^, ^39^ may result in higher charge capacities in the first cycle. First discharge capacities of 377, 409, 362, 375 mAh g^−1^ were obtained for *x* = 0, 0.2, 0.5, 0.8 samples, respectively. The results indicate that about 1.57–1.77 Li^+^ per unit formula can be reversibly delivered in/out of the host structure. The highest specific energy (≈1140 Wh kg^−1^) at 13 mA g^−1^ and 25 °C was obtained for *x* = 0.2. When the samples cycled at 525 mA g^−1^ (about 1.1 C) and 40 °C, reversible capacities of about 345 mAh g^−1^ (1.5 Li^+^) were still accessible for *x* = 0.2 (Figure 3e). In comparison, pure chromium oxyfluoride (*x* = 0) delivered a reversible capacity of ≈284 mAh g^−1^. Note that clear difference in the discharge curves between *x* = 0.2 and *x* = 0 was observed (Figure 3e). The discharge curves above 2.4 V between two samples are overlapped, whereas extended discharge profile below 2.4 V was seen for *x* = 0.2, arising from the contribution of further vanadium reduction process. Nevertheless, both materials (*x* = 0 and 0.2) were still able to deliver impressively high specific energies of ≈763 and ≈960 Wh kg^−1^ at ≈1 C rate, respectively, in comparison to 630 Wh kg^−1^ in LiNi_1/3_Mn_1/3_Co_1/3_O_2_ 40 and 840 Wh kg^−1^ in layered (1−*x*)LiMO_2_·*x*Li_2_MnO_3_ (M = Ni, Co, Mn) cycled at low rate (≈C/24).^11^ The cyclic stability of Li_2_V*_x_*Cr_1−*x*_O_2_F showed higher capacity retention for the mixed V and Cr oxyfluorides (Figure 3f), compared to their end members.^33^ The best capacity retention was observed for *x* = 0.5. After 60 cycles, 70% of the capacity remained, in comparison to ≈50% for pure chromium oxyfluoride.

**Figure 3:**
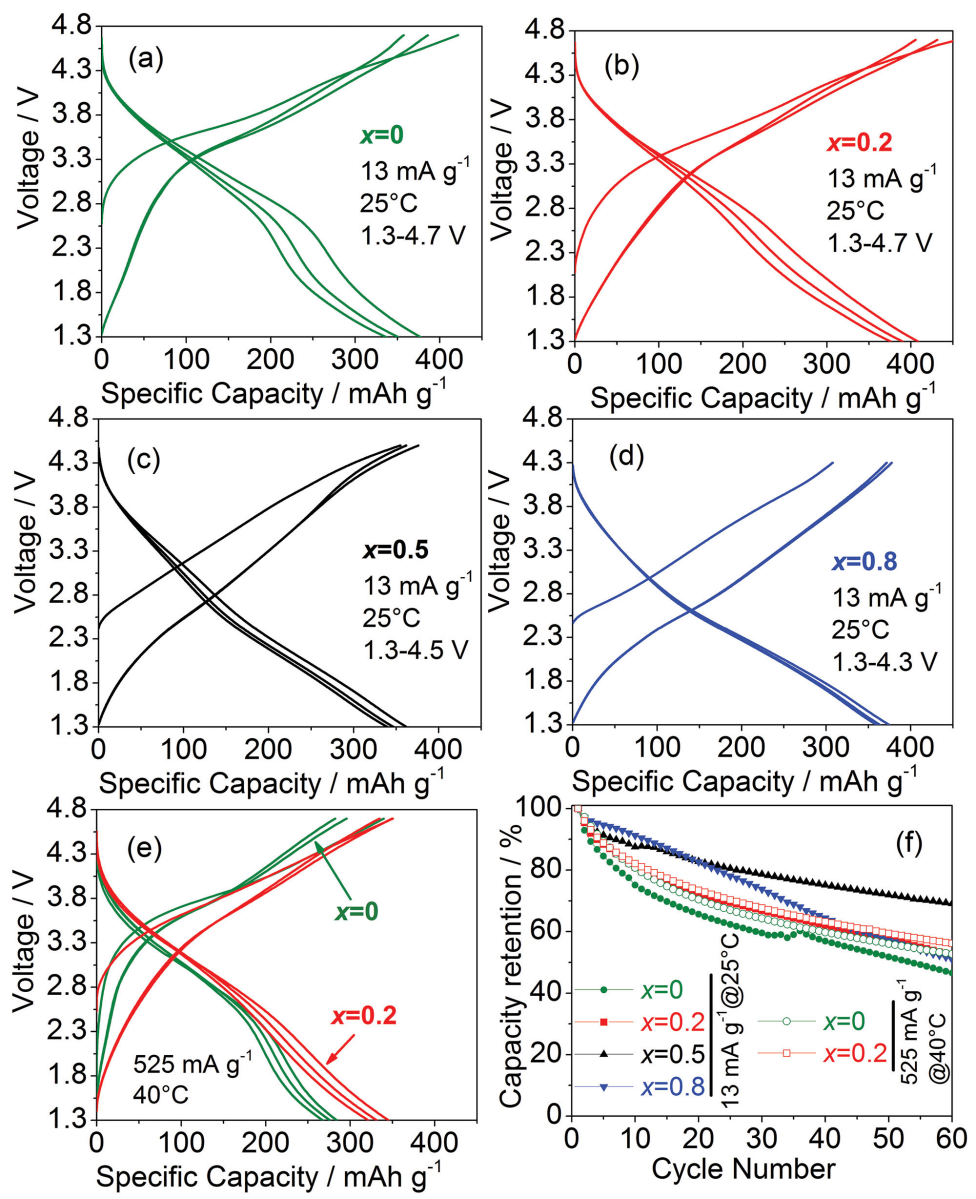
Charge/discharge curves in first three cycles of a–d) Li_2_V*_x_*Cr_1−*x*_O_2_F (*x* = 0, 0.2, 0.5, 0.8, respectively) at 13 mA g^−1^ and 25 °C in varied voltage ranges and of e) *x* = 0 and *x* = 0.2 at 525 mAh g^−1^ at 40 °C in the voltage range of 1.3–4.7 V. f) The corresponding discharge capacity retentions for all tested samples in (a)–(e).

The battery performance was further evaluated with varied charge cutoff voltages (**Figure** **4** and Figure S7, Supporting Information). Higher cutoff voltage led to obvious increase in the charge and discharge capacities. For *x* = 0.5, the discharge capacities increased from 300 to 362 mAh g^−1^ when charge cutoff voltages were set from 4.1 to 4.5 V, respectively. Similarly, additional 0.3 Li^+^ per unit formula was extracted for *x* = 0.8 with a charge cutoff voltage increase of 0.4 V. For the vanadium‐dominated samples, the cell cannot be cycled well when higher cutoff voltages are applied. This may relate to side reactions or structural change under high voltages.^37^, ^38^, ^39^ Nevertheless, enhanced lithiation voltages were clearly observed when higher cutoff voltage was applied, especially in the case of *x* = 0.5 (Figure 4a). For *x* = 0.5, the discharge curves shifted up by about 0.3 V with cutoff voltage of 4.5 V compared to 4.1 V. This indicates the contribution of chromium redox reaction at high voltages. However, higher cutoff voltages caused lower cyclic stability (Figure 4c), which may relate to electrolyte decomposition and surface instability of the transition metals under high voltages.^37^, ^38^, ^39^ To maximize the accessible capacities and complete redox capability of V and Cr in the studied chromium‐dominated samples (*x* = 0.2 and *x* = 0), both samples were cycled between 1.3 and 4.7 V.

**Figure 4:**
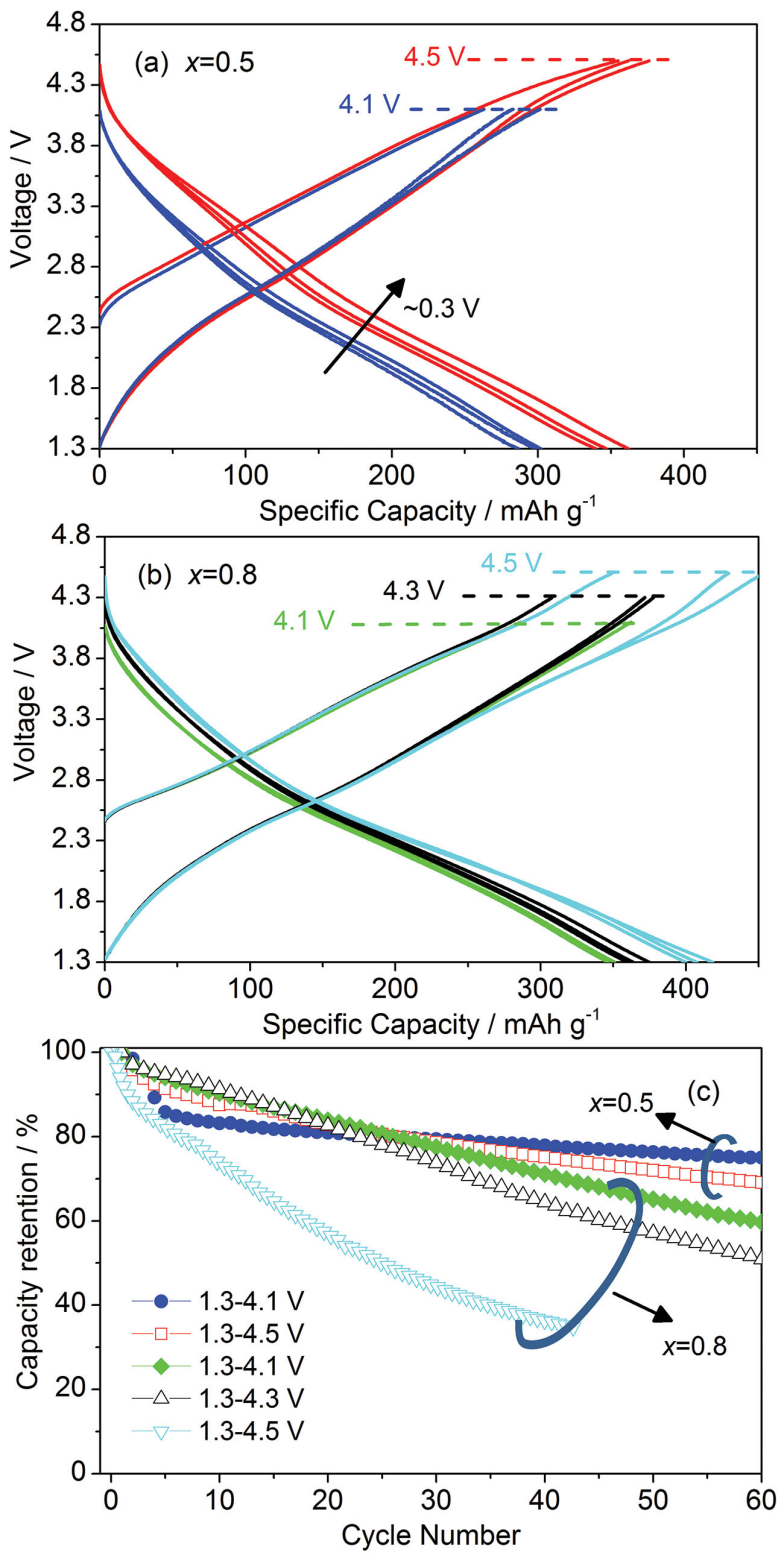
Charge/discharge profiles of a) *x* = 0.5 and b) *x* = 0.8 under varied cutoff voltages at 13 mA g^−1^ and 25 °C; c) discharge capacity retentions in (a) and (b).

In order to reveal structural changes upon charge/discharge process, various characterization methods were used on the corresponding charged/discharged samples in comparison to the pristine sample. For *x* = 0.2, ex situ SXRD (Figure 1a) showed that the diffraction peaks of *Fm*‐3*m* shifted to higher angle after charge to 4.7 V and shifted back after discharge. This corresponds to a minor volume change of ≈3% (for ≈1.77 Li^+^ intercalation), implying a reversible structure change upon cycling in a rigid host structure with low strain involved. Interestingly, after removal of 1.77 Li^+^, the disordered structure still remained. The impurity phases as WC and Cr_2_O_3_ stayed unchanged over charge/discharge process. No evidence of any new phase formation during electrochemical process was found. Cr K‐edge XANES was used to examine chromium oxidation and local structure change upon cycling for both *x* = 0 and 0.2 (**Figure** **5** and Figure S4, Supporting Information). Upon charge, the absorption edge shifted to higher energy due to the oxidation of Cr. Meanwhile, the presence of a weak pre‐edge peak was clearly observed, indicating certain distortion from octahedral in local structure upon delithiation.^41^, ^42^ Upon discharge, the absorption edge shifted back to lower energy whereas the pre‐edge peak vanished, indicating the average oxidation state of chromium converted back. After second charge, similar behavior was observed compared to that in the first charge process. The intensity of the pre‐edge peak was almost the same as that after first charge. The shape of overall near‐edge spectra changed reversibly upon charging and discharging, suggesting reversible redox and structure change. Note that chromium is electrochemically inactive in the layered rock salt structure (*R*‐3*m*) such as LiCrO_2_.^43^, ^44^, ^45^ Cr substitution in layered LiVO_2_ leads to improved cyclic stability but Cr stays inactive in electrochemical process.^43^ The capability to tune electrochemical performance of chromium was reported by atom substitutions in solid solution of LiMn*_x_*Cr_1−*x*_O_2_.^44^ In our case, chromium in disordered rock salt structure exhibited its distinct electrochemical activity. The reversible ≈1.77 Li^+^ intercalation in *x* = 0.2 corresponds to the average valence change from +3 to +4.77 by combining both V and Cr redox couples. As a further proof for both Cr and V oxidation in charged sample for *x* = 0.2, energy loss near edge structure (ELNES) spectra for the pristine and charged *x* = 0.2 are shown in Figure 5b. It clearly evidenced that V L_3_, L_2_‐edges^46^ and Cr L_3_, L_2_‐edges^47^ shifted to higher energy loss upon charging, confirming oxidation of both V and Cr after electrochemical Li^+^ extraction.

**Figure 5:**
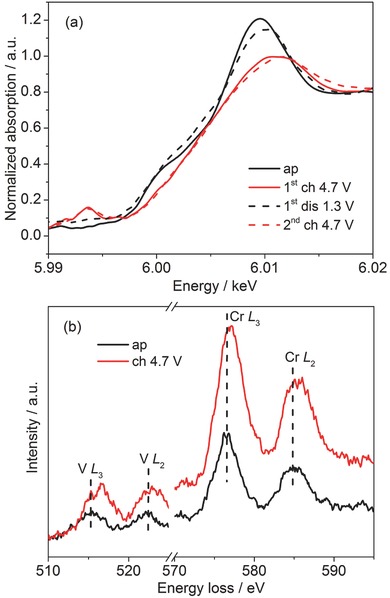
a) Normalized Cr K‐edge XANES spectra and b) typical L_3,2_ ELNES for as‐prepared Li_2_V_0.2_Cr_0.8_O_2_F and samples after first charge, first discharge, and second charge.

Disordered structure for both as‐prepared and charged samples was also evidenced by HR‐TEM and the corresponding line profiles (Figure S8, Supporting Information). All atoms of Cr, V, F, and O in both as‐prepared and charged samples were identified by energy‐dispersive spectroscopy (EDS) (Figure S8, Supporting Information). These results suggested that the rigid framework based on disordered cubic rock salt for dilithium metal oxyfluoride stabilized well upon lithium extraction/reinsertion. For layered oxide materials, migration of cations between transition metal layers and Li layers during electrochemical cycling caused increased trapping of metal ions in interstitial tetrahedral sites.^12^ However, such migration has not been evidenced in our case. Further work is ongoing to clarify the local structural features of the materials upon Li^+^ removal in the DRS structure.

In conclusion, a solid‐solution series of Li_2_V*_x_*Cr_1−*x*_O_2_F having disordered cubic rock salt structure with a space group of *Fm*‐3*m* was synthesized by a simple ball‐milling route. The work demonstrates the feasibility to increase the cell voltage and enhance the cyclability for the high‐capacity disordered oxyfluoride materials by simple cation substitutions. About 1.6–1.8 Li^+^ per unit formula can be reversibly stored in the low strain host materials, corresponding to attractively high specific capacities of 360–410 mAh g^−1^. The rate performance shows that a high specific energy of 960 Wh kg^−1^ is still accessible at ≈1 C rate for *x* = 0.2. Such materials may be alternative contenders to the existing intercalation cathode materials. Further work by introducing environmentally benign and cost‐effective elements into such disordered oxyfluoride structure for practical application considerations is going on. These findings are expected to inspire new activities in developing high energy/power oxyfluoride intercalation cathode materials and open up a new avenue to the search for advanced energy storage materials.

## Supporting information

As a service to our authors and readers, this journal provides supporting information supplied by the authors. Such materials are peer reviewed and may be re‐organized for online delivery, but are not copy‐edited or typeset. Technical support issues arising from supporting information (other than missing files) should be addressed to the authors.

Supplementary
