# Supplementary material for: Improved Voltage and Cycling for Li+ Intercalation in High‐Capacity Disordered Oxyfluoride Cathodes
Source: Adv Sci (Weinh). 2015 Jun 12;2(10):1500128. doi: 10.1002/advs.201500128 (PMC5115328; doi:10.1002/advs.201500128)
Supplement: Supplementary file 1 — Supplementary [file ADVS-2-0m-s001.pdf]

Copyright WILEY-VCH Verlag GmbH & Co. KGaA, 69469 Weinheim, Germany, 2015.

ADVANCED SCIENCE
Open Access

Supporting Information

for *Adv. Sci.*, DOI: 10.1002/advs.201500128

Improved Voltage and Cycling for Li^+ Intercalation in High-Capacity Disordered Oxyfluoride Cathodes

Shuhua Ren, Ruiyong Chen,* Emad Maawad, Oleksandr Dolotko, Alexander A. Guda, Viktor Shapovalov, Di Wang, Horst Hahn, and Maximilian Fichtner*

WILEY-VCH

Copyright WILEY-VCH Verlag GmbH & Co. KGaA, 69469 Weinheim, Germany, 2013.

Supporting Information

Improved Voltage and Cycling for Li^+ Intercalation in High-Capacity Disordered Oxyfluoride Cathodes

Shuhua Ren, Ruiyong Chen,* Emad Maawad, Oleksandr Dolotko, Alexander A. Guda, Viktor Shapovalov, Di Wang, Horst Hahn and Maximilian Fichtner*

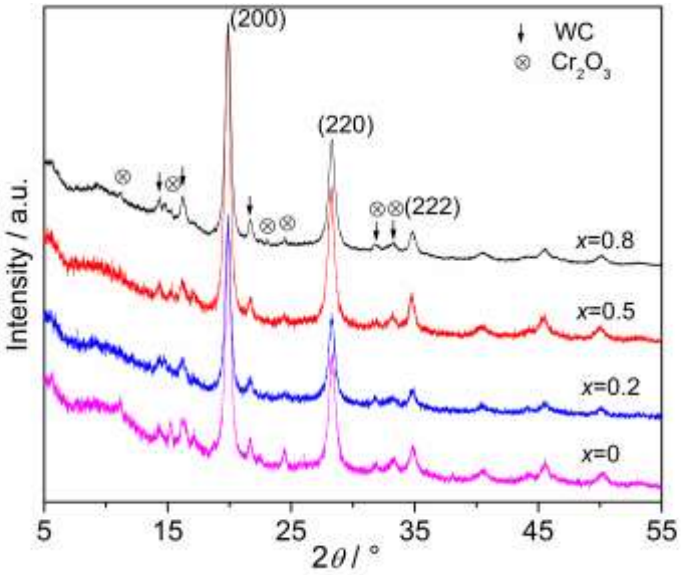

Figure S1. XRD patterns (Mo Kα_1, λ = 0.70932 Å) of the as-prepared $Li_2V_xCr_{1-x}O_2F$ (x = 0, 0.2, 0.5, 0.8).

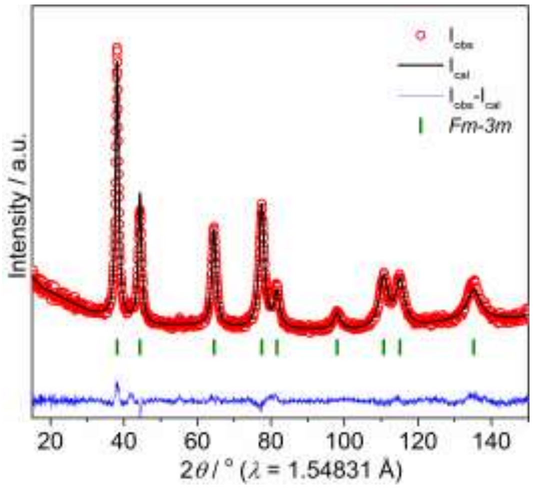

Figure S2. Neutron diffraction pattern of $Li_2V_{0.2}Cr_{0.8}O_2F$ with the observed data (red dots), calculated pattern (black line), difference plot (blue line) and the calculated Bragg positions. R_p = 15.1, R_{wp} = 11.6, R_e = 8.88.

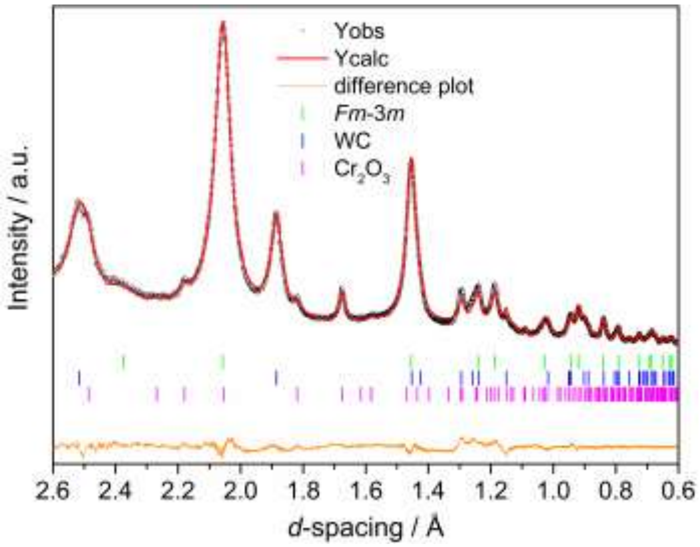

Figure S3. Synchrotron X-ray diffraction (λ = 0.41343 Å) of the $Li_2V_{0.2}Cr_{0.8}O_2F$ with the observed data (black dots), calculated pattern (red line), difference plot (orange line) and calculated Bragg positions. R_p = 9.41, R_{wp} = 10.5, R_e = 7.57.

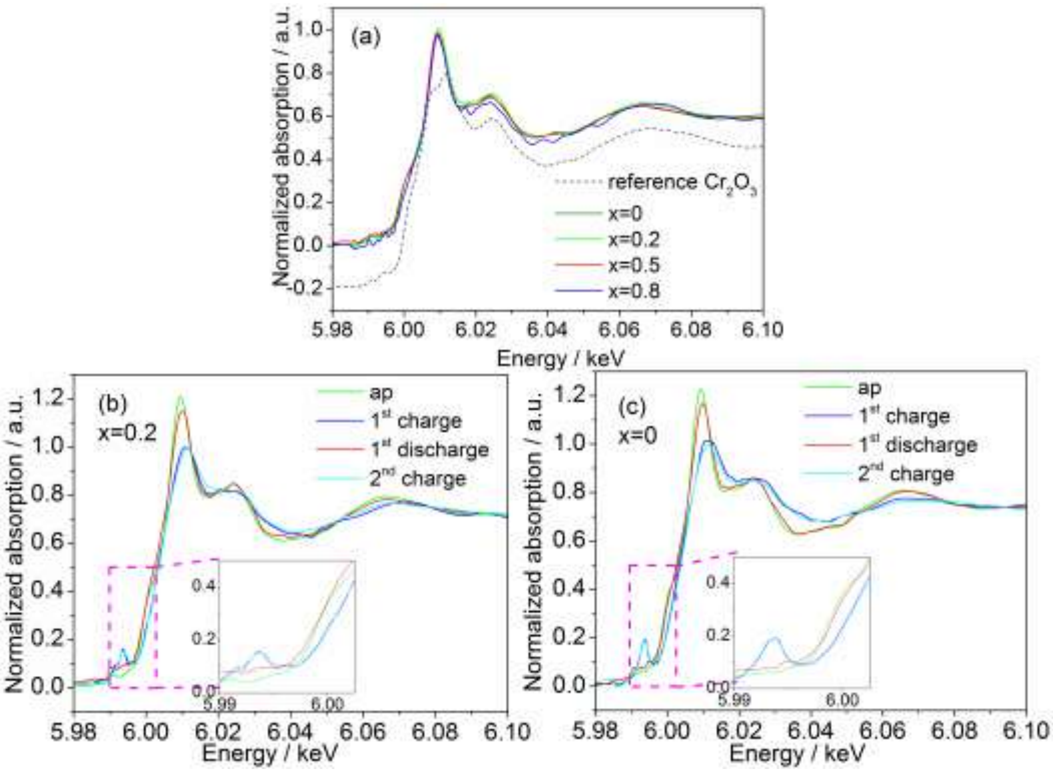

Figure S4. Normalized Cr *K*-edge XANES spectra of (a) as-prepared $Li_2V_xCr_{1-x}O_2F$ (x = 0, 0.2, 0.5, 0.8). Cr_2O_3 as reference, shifted for better clarification; (b) as-prepared (x = 0.2) and (c) (x = 0) and their corresponding samples after 1st charge, 1st discharge and 2nd charge.

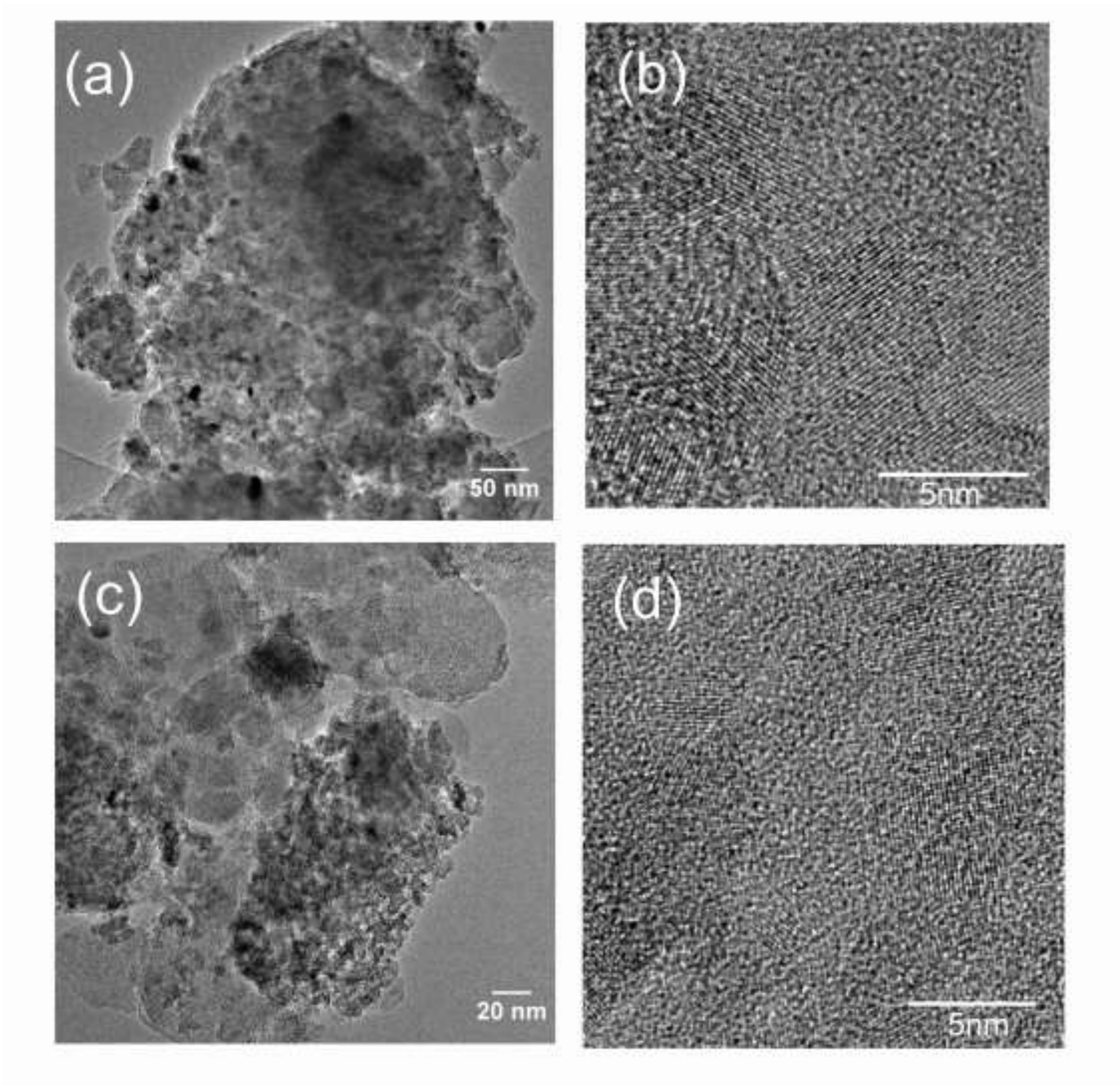

Figure S5. TEM of (a, b) as prepared $Li_2V_{0.2}Cr_{0.8}O_2F$ and (c, d) the sample charged to 4.7 V.

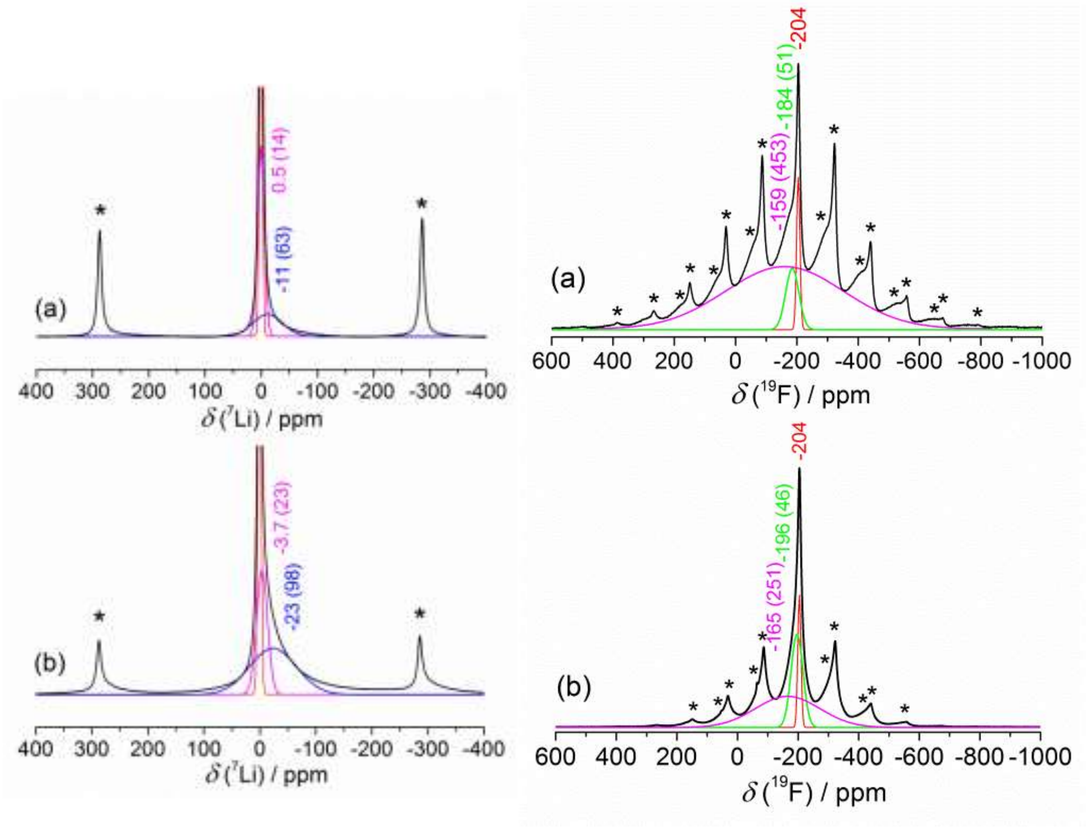

Figure S6. ^{7}Li and ^{19}F NMR spectra of (a) x = 0.2 and (b) x = 0.8. The * denotes the spinning sidebands. The Line width values were given in parentheses.

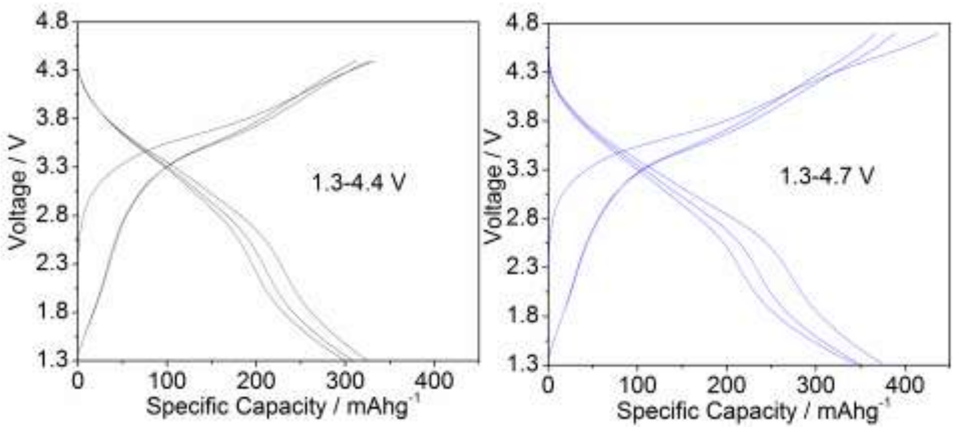

Figure S7. Charge-Discharge profiles in the first three cycles for (x=0) under varied cut-off voltages at 26 mA g^{-1} and 25°C.

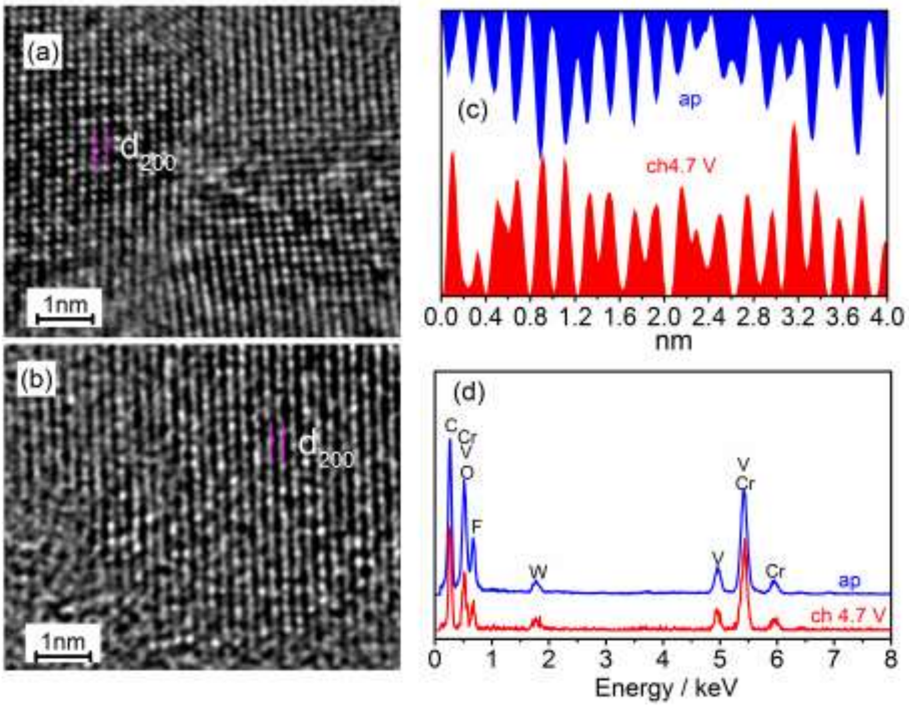

Figure S8. HRTEM images of (a) as-prepared $Li_2V_{0.2}Cr_{0.8}O_2F$ sample and (b) sample charged to 4.7 V; (c) the representative line profiles; (d) EDS spectra.

Experimental Section

Synthesis. $Li_2V_xCr_{1-x}O_2F$ (x = 0.2, 0.5, 0.65, 0.8, 1.0) powders were synthesized by a planetary-type ball-milling (450 rpm for 40 h, Fritsch Pulverisette 6, WC milling jar and balls) using Li_2O (10% excess), LiF, V_2O_3 and Cr_2O_3 as precursors. Precursors mixture were added into WC jar in an argon-filled glovebox. After ball-milling, the powders were taken out inside the glovebox and stored under an argon atmosphere.

Neutron diffraction. Neutron diffraction (ND) experiments were carried out at room temperature using the high resolution powder diffractometer SPODI [s1] at the neutron source FRM II at the Hans Maier Leibnitz Zentrum, Garching. The powder sample of about 1 g was sealed in cylindrical vanadium container with a diameter of 6 mm and investigated in transmission mode. The integration time for each diffraction pattern was 6 hours. The measurements were performed using monochromatic neutrons with λ = 1.54831(2) Å obtained from a vertically focused composite Ge (551) monochromator. The vertical position-sensitive multi-detector of 300 mm effective height consists of 80 ^{3}He tubes and covers an angular rang of 2θ from 0° to 160°. All measurements were performed in Debye-Scherrer geometry with an incident neutron beam having a rectangular cross section at the sample position of 40×20 mm^2.

X-ray diffraction. Powder X-ray diffraction (XRD) data were collected on a STOE Stadi/P diffractometer with Mo $K\alpha_1$ radiation (λ = 0.70932 Å). Synchrotron X-ray diffraction (SXRD) data were collected at the MSPD beamline (λ = 0.41343 Å) at the ALBA Synchrotron Light Source, Spain. The powders were loaded into 0.7 mm glass capillaries in an argon-filled glovebox. The diffraction profiles were refined using the Rietveld method.

TEM and NMR. Transmission electron microscopy (TEM) images and electron energy-loss near edge structure (ELNES) spectra were acquired using a Titan 80-300 microscope (FEI,

US). Solid-state ^{7}Li, ^{19}F-magic angle spinning (MAS) nuclear magnetic resonance (NMR) experiments were performed on a Bruker Advance Spectrometer with a 1.8 mm MAS probe at room temperature with adjusted ^{7}Li (90°/180° pulses of 2.0/4.0 μs), ^{19}F (0.8/1.6 μs pulse lengths).

X-ray absorption. The chromium *K*-edge X-ray absorption near edge structure (XANES) spectra were recorded using an in-house Rigaku R-XAS spectrometer in transmission mode at room temperature with a crystal monochromator Ge(311) and an energy resolution of 0.7 eV, at the Southern Federal University, Russia. The samples were pressed in pellets and sealed in an air-tight plastic bag under inert atmosphere. Ten spectra were acquired and averaged for each sample.

Electrochemistry. For electrochemical analysis, the as-obtained $Li_2V_xCr_{1-x}O_2F$ powder was mixed with 30% Super P and ball-milled at 450 rpm for 10 h. Li foil was used as counter electrode. 1 M $LiPF_6$ in ethylene carbonate/dimethyl carbonate (EC/DMC, 1:1 v/v) was used as electrolyte. The working electrode consisted of ~85 wt% of $Li_2V_xCr_{1-x}O_2F$/Super P composite and ~15 wt% of poly(vinylidene fluoride hexafluoropropylene) (PVDF-HFP) copolymer (Solef 5130). Electrochemical measurements were carried out using Swagelok-type cells in the varied voltage range between 1.3 V and 4.7 V at different temperatures (40°C, 25°C) and varied current rates of 13–525 mA g^{-1}.

Reference

[s1] M. Hoelzel, A. Senyshyn, N. Juenke, H. Boysen, W. Schmahl and H. Fuess, High-resolution neutron powder diffractometer SPODI at research reactor FRM II, *Nucl. Instr. Meth. A,* 2012, **667**, 32-37.
